# Remote Delivery of Yoga Interventions Through Technology: Scoping Review

**DOI:** 10.2196/29092

**Published:** 2022-06-06

**Authors:** Aurora James-Palmer, Ellen Zambo Anderson, Jean-Francois Daneault

**Affiliations:** 1 Department of Rehabilitation and Movement Sciences Rutgers University Newark, NJ United States

**Keywords:** complementary therapies, mind-body, remote delivery, telerehabilitation, eHealth, yoga, technology, mind-body

## Abstract

**Background:**

The popularity of yoga and the understanding of its potential health benefits have recently increased. Unfortunately, not everyone can easily engage in in-person yoga classes. Over the past decade, the use of remotely delivered yoga has increased in real-world applications. However, the state of the related scientific literature is unclear.

**Objective:**

This scoping review aimed to identify gaps in the literature related to the remote delivery of yoga interventions, including gaps related to the populations studied, the yoga intervention characteristics (delivery methods and intervention components implemented), the safety and feasibility of the interventions, and the preliminary efficacy of the interventions.

**Methods:**

This scoping review was conducted in accordance with the PRISMA-ScR (Preferred Reporting Item for Systematic Reviews and Meta-Analyses extension for Scoping Reviews) guidelines. Scientific databases were searched throughout April 2021 for experimental studies involving yoga delivered through technology. Eligibility was assessed through abstract and title screening and a subsequent full-article review. The included articles were appraised for quality, and data were extracted from each article.

**Results:**

A total of 12 studies of weak to moderate quality were included. Populations varied in physical and mental health status. Of the 12 studies, 10 (83%) implemented asynchronous delivery methods (via prerecorded material), 1 (8%) implemented synchronous delivery methods (through videoconferencing), and 1 (8%) did not clearly describe the delivery method. Yoga interventions were heterogeneous in style and prescribed dose but primarily included yoga intervention components of postures, breathing, and relaxation and meditation. Owing to the heterogeneous nature of the included studies, conclusive findings regarding the preliminary efficacy of the interventions could not be ascertained.

**Conclusions:**

Several gaps in the literature were identified. Overall, this review showed that more attention needs to be paid to yoga intervention delivery methods while designing studies and developing interventions. Decisions regarding delivery methods should be justified and not made arbitrarily. Studies of high methodological rigor and robust reporting are needed.

## Introduction

### Background

As of 2016, a total of 36 million Americans had engaged in some form of yoga practice [1], with other countries demonstrating similar patterns of yoga practice [2-4]. Although healthy individuals incorporate yoga into their fitness routines to improve physical and mental health [1], yoga has also been used to manage symptoms of disease [2-5]. Even before the COVID-19 pandemic in 2020, individuals sought access to yoga in their home environments. In fact, results from a 2016 survey revealed that yoga was most commonly practiced at home [1]. During the pandemic, public health policies and individuals’ personal preference for social distancing and staying at home stopped or limited in-person practice. Under these circumstances, the availability of resources to practice yoga remotely increased, which could be beneficial for individuals with functional limitations.

However, when examining the available scientific literature, yoga interventions are most often delivered in person [6]. Although this may be feasible for constrained research projects, it greatly limits real-world applicability. First, access to qualified yoga instructors inevitably varies based on geographical location [7]. Second, individuals’ socioeconomic status and access to transportation limit their access [8]. Third, inherent physical limitations may hinder the patients’ ability to attend in-person yoga classes. Therefore, in-person yoga instruction presents several barriers regardless of the public health climate, such that alternative delivery methods (eg, videos, videoconferencing, and mobile apps) have been developed and are being used extensively. However, there seems to be a gap in the literature regarding interventions conducted in research studies and real-world practices.

Little is known about the evidence regarding the practice and outcomes of yoga using remote delivery methods. A previous scoping review of the yoga literature [6] included studies in which yoga was conducted at sites other than yoga studios, such as *at home*, where yoga instructors may not be present. However, details regarding intervention delivery were not provided, although there are multiple ways of delivering yoga remotely [6]. For example, information about yoga can be provided through hard copy resources, such as pamphlets and card decks, or through more engaging technological means. Specifically, technologies such as videoconferencing, DVDs, websites, and mobile apps can be used to provide yoga interventions. Furthermore, content can be delivered either (1) synchronously (an instructor interacts with participants in real time, eg, through videoconference) or (2) asynchronously (material is prerecorded for participants to use without real-time interaction). Despite these options and their potential impact, few studies have explored their use. Thus, little is known about how yoga is delivered remotely, whether remote delivery is used for some specific populations more than others, and what types of yoga are being delivered remotely.

With this, it is important to know that yoga is a multilayered ancient philosophy and practice intended to facilitate well-being through the cultivation of awareness by integrating mind and body, with an emphasis on self-realization [9]. There are various forms, often referred to as branches of yoga, which facilitate one’s ability to reach a greater state of being. For instance, *Karma yoga* is the branch that focuses on devotion to service, *Jnana yoga* focuses on the development of knowledge, and *Hatha yoga* involves the practice of physical postures. *Hatha yoga* is typically what comes to mind when one thinks of *yoga* in the Western world. The term *Hatha yoga* refers to the branch of the physical practice of yoga and is also usually used to refer to a broad style of yoga that incorporates postures and breathing. There are other styles of yoga that fit under the umbrella of the physical practice of yoga—*Hatha yoga*—but are often specified further*.* Examples of these styles include *Iyengar yoga* and *Vinyasa yoga.* For example, *Iyengar yoga* focuses on body alignment, sequencing, and timing. By contrast, *Vinyasa yoga* is generally energetic and involves flowing through sequences of postures with breath integration. In addition to the branches of yoga and specific styles of the physical practice of yoga, there are 8 limbs of yoga. These 8 limbs are described in the *Yoga Sutras of Patanjali*, a foundational text, and include (1) *yama* (abstinences), (2) *niyama* (observances), (3) *asanas* (postures), (4) *pranayama* (breath control), (5) *pratyahara* (withdrawal of the senses), (6) *dharana* (concentration), (7) *dhyana* (meditation), and (8) *samadhi* (bliss) [9]. These limbs help to inform the practice of yoga in general, and some of them are incorporated in *Hatha yoga.* Most commonly, *asana*, *pranayama*, and *dhyana* are incorporated into *Hatha yoga*; however, in some cases, different combinations of the limbs of yoga are used. A previous scoping review of the yoga literature showed that most yoga intervention studies did not specify the style of yoga used however, of those that did, the most commonly reported styles were *Hatha yoga* (129/456, 28.3% of studies) and *Iyengar yoga* (41/456, 8.9% of studies) [6]. It is unknown whether similar patterns occur when yoga is delivered remotely.

### Objective

Yoga can be delivered both in person and remotely, and there has been an increase in the availability and use of remotely delivered interventions in real-world applications. However, it is not known whether the scientific literature reflects this or reflects what types of populations have been enrolled in studies that have investigated the remote delivery of yoga, what the characteristics of these interventions are (including how remote delivery occurs), and what components of yoga are incorporated. Furthermore, the general feasibility and safety of these interventions or their preliminary efficacy are yet to be clearly defined. Therefore, the purpose of this scoping review was to examine the existing literature regarding the practice of yoga through remote delivery methods and identify current gaps related to (1) the populations studied, (2) the intervention characteristics (delivery methods and intervention components implemented), (3) the safety and feasibility of the interventions, and (4) the preliminary efficacy of the interventions.

## Methods

This review was conducted in accordance with the PRISMA-ScR (Preferred Reporting Items for Systematic Reviews and Meta-Analyses extension for Scoping Reviews) guidelines [10].

### Search Strategy

Scientific databases, including PubMed, Scopus, Web of Science, PEDro, CINAHL, PsycINFO, IEEE Xplore Digital Library, and Cochrane Library, were searched during April 2021. The search strategies were modified for each database, with the Medical Subject Headings terms used as applicable. The following terms were used in various combinations: *yoga*, *Iyengar*, *ashtanga*, *hatha*, *asana*, *telerehabilitation*, *tele-rehabilitation*, *telemedicine*, *videoconferencing*, *video*, *telenursing*, *DVD*, *remote delivery*, *eHealth*, *video games*, *online*, and *virtual reality* (see Multimedia Appendix 1 for the specific search terms and search methods used for each database).

### Inclusion and Exclusion Criteria

Articles were included if they investigated (1) adults aged ≥18 years; (2) an experimental intervention study with pre- and posttesting for at least one group; (3) a yoga intervention using physical yoga postures (*asanas)* and at least one other of the [9] 8 limbs of yoga, as described in the *Yoga Sutras of Patanjali,* including *yama* (abstinences), *niyama* (observances), *pranayama* (breath control), *pratyahara* (withdrawal of the senses), *dharana* (concentration), *dhyana* (meditation), and *samadhi* (bliss); and (4) a method of remote yoga delivery through technology for at least one group (eg, video, mobile app, and videoconferencing platform) as the primary intervention.

Yoga interventions can be heterogeneous and are often poorly described in the literature [6]. In some cases, they do not reflect the integration of the mind and body as intended in traditional practice [9]. To address this, we stipulated that selected articles should explicitly include physical postures (body) and another of the [9] 8 limbs of yoga (mind) using either English or Sanskrit words. This was intended to limit poorly described interventions or interventions in which yoga was not the primary intervention. For example, although mindfulness-based stress reduction includes components of yoga, there are other significant intervention components that make these interventions different from typical yoga interventions, which, if included, introduce even more heterogeneity across studies. Furthermore, it also allowed us to exclude studies that used only physical postures that could be more akin to exercise interventions.

### Article Screening and Data Extraction

All the data management processes discussed in this section were completed by the 3 authors (AJP, EZA, and JFD) in the following manner. The articles were divided into thirds. Then, 2 authors, screened, reviewed, and appraised, (two-thirds or 66.7% of the articles) with the third author available to resolve conflicts, such that each author (AJP, EZA, and JFD) was able to perform each task. The abovementioned criteria guided eligibility screening (completed via Rayyan QCRI [11]) and full-text article assessment. For articles that were included based on the full-text assessment, data were extracted simultaneously with the full-article review, and then a quality assessment was completed. The Quality Assessment Tool for Quantitative Studies Effective Public Health Practice Project was used to assess the level of bias and methodological quality of each study [12]. Data were extracted into a Microsoft Excel sheet designed by the scoping review authors (initial design by AJP, edited and approved by EZA and JFD) before the article review. The data extraction categories were determined by the authors (initial selection by AJP, edited and approved by EZA and JFD) based on the objective of the review (see Table 1 for descriptions and examples of data extracted from each study).

**Table 1:** Data extracted from selected articles (scoping review; information extracted from each selected article and examples).

Content area and extracted information			Examples
**General study information**			
	Study type as defined by the study authors in the introduction or methods sections	Randomized controlled trial; single-group study	
	Country in which the study was conducted	United States	
	Comparison group used as described by the study authors (if applicable)	Regular activity control group; active control group such as a strengthening program	
**Study populations**			
	Number of participants (total [N] and per group [n])	N=50	
	Description of population, including defining characteristics such as the health condition, as described by the study authors	Women with depression	
	Mean age of the participants and SD (if provided) for the total sample and each group	Mean age of the total sample was 55.07 (SD 9.69) years	
	Sex distribution of participants in the total sample and in each group	Number of women in the study out of the total number of participants	
	Justification of delivery method in relation to the study population, as described by the study authors (ie, did the study authors describe why they delivered the intervention remotely, and if they did, what was the reason)	Yes—the study authors reported that individuals with cancer often have transportation and scheduling challenges that make it difficult to attend in-person appointments; no—the study authors did not describe why they chose remote delivery	
**Intervention characteristics: delivery methods**			
	Intervention setting	Home	
	Whether delivery was synchronous or asynchronous; delivery was considered synchronous if interventions were delivered in real time such that the instructor could interact with the participant or participants; delivery was considered asynchronous if intervention materials were prerecorded and could be accessed at any time	Synchronous (videoconferencing) and asynchronous (prerecorded video)	
	The technology used to deliver the intervention	Name of a specific videoconferencing platform; type of prerecorded video (ie, DVD)	
	Whether delivery was group or individual; it was considered group delivery if multiple people participated in the yoga intervention together at one time; it was considered individual delivery if a participant engaged in the intervention alone	If each participant received access to a prerecorded video and watched the video on their own (individual delivery)	
	Whether participants had additional interactions with the study team outside of assessment sessions and prescribed intervention sessions	Participants received an in-person introduction yoga class before starting the intervention period	
	Whether participants received supplementary materials	Participants received written instructions providing additional information on how to practice yoga	
**Intervention characteristics: yoga intervention components and other details**			
	Style of yoga implemented	Hatha yoga or Iyengar yoga	
	Specific limbs of yoga implemented	Breathing; postures; meditation; relaxation	
	Yoga instructor credentials, as reported by the study authors, including instructor training (ie, are they a yoga instructor, yoga therapist, or other health care professional) and their certification training hours	Yoga instructor (200 hours)	
	Yoga dose: frequency and duration reported in minutes per session, sessions per week, and total number of weeks	30 minutes per session with 2 sessions per week for 6 weeks	
	Whether the yoga sequences were designed, adapted, or selected for the specific study population, as described by the study authors, or whether this was not reported	Yes—the study authors reported that they designed the prerecorded videos specifically for the population enrolled in the study; no—the study authors did not report whether the yoga intervention was designed for the study population	
	Information about additional home practice (ie, did the study authors describe whether participants were encouraged to engage in additional practice outside of the prescribed intervention and how this was kept track of)	Yes—although the study authors required participants to watch the yoga video 1 time per week, the study authors encouraged participants to view the yoga video an additional 2 to 3 times per week if possible and asked them to log how often they did this	
**Intervention feasibility and safety**			
	Study adherence (ie, did participants complete the study overall, including the intervention period and assessment sessions) reported as how many people in each group completed the study	66% (44/67) of the yoga group completed the study	
	Intervention adherence (ie, did participants complete the intended yoga intervention dose); intervention adherence was reported as it was reported in each study; some studies reported it as the mean yoga practice, whereas others set a threshold or benchmark and reported intervention adherence as it related to the benchmark	Mean yoga practice was 44 min/week and the prescribed dose was 60 min/week; the benchmark for “good adherence” was participants who practiced yoga ≥6 times over 2 weeks, and 55% (37/67) of the yoga group met this benchmark	
	The presence or absence of technological challenges, as described by the study authors	Participants experienced technological challenges in 77% (24/31) of the sessions	
	The presence or absence of adverse events, as reported by the study authors for each study group, or whether the study authors did not report any information about adverse events	No adverse events occurred, the study did not report information about adverse events; 9 mild adverse events occurred in the yoga group and 4 mild adverse events occurred in the comparison group	
**Preliminary efficacy**			
	The outcome measures were categorized into patient-reported outcome measures, physical performance and function outcome measures, and physiological outcome measures based on what the measures assessed; subsequently, a summary of results for these outcomes was extracted (eg, were there significant improvements between groups and significant improvements within groups)	The patient-reported outcome measure—the Beck Depression Inventory—showed significant within-group improvements	

## Results

### Overview

**Figure 1:**
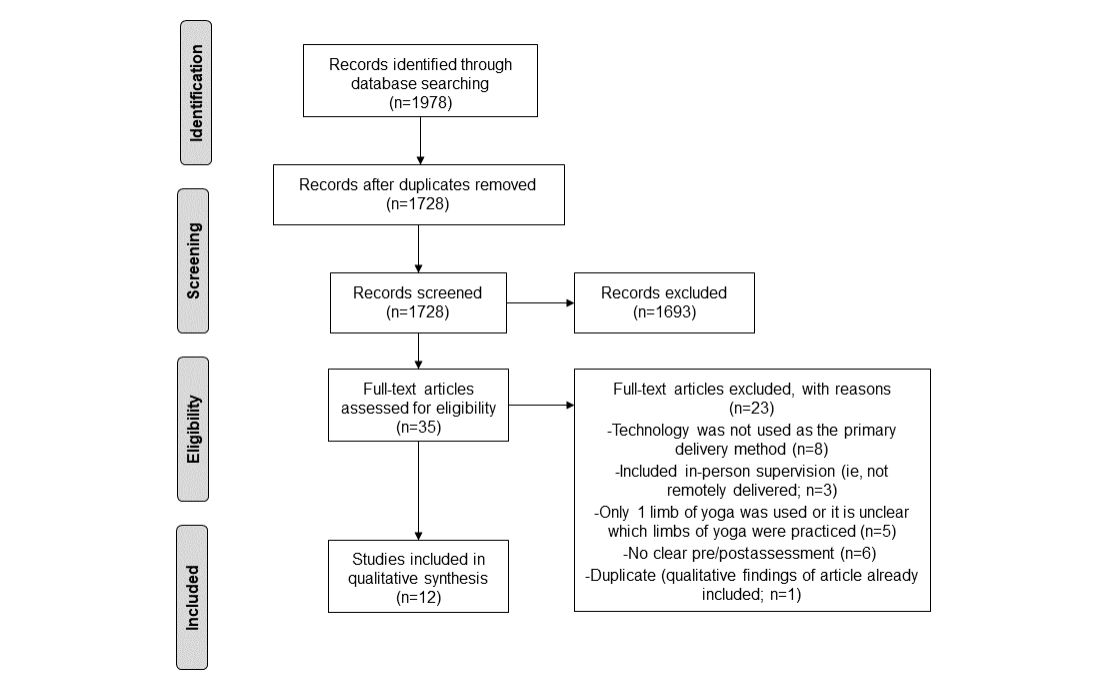
Illustration showing the PRISMA (Preferred Reporting Items for Systematic Reviews and Meta-Analyses) flow diagram of the search, screening, and full-article review results.

The search resulted in 1978 articles, with 1728 (87.36%) remaining after duplicates were removed. The title and abstract screening resulted in the inclusion of 2.03% (35/1728) of articles. Following a full-article review, of the 35 articles, 12 (34%) articles [13-24] published between 2003 and 2021 were included in the final qualitative analysis (see Figure 1 for the PRISMA [Preferred Reporting Items for Systematic Reviews and Meta-Analyses] flowchart).

### General Study Information and Study Quality

Of the 12 studies, 8 (67%) were randomized controlled trials (RCTs) [13,14,18-23], 2 (17%) were quasi-experimental nonrandomized studies [16,17], 1 (8%) was an open-label single-group study [24], and 1 (8%) was a case series [15]. The comparison groups in the RCTs included regular activities [14,21], wait-list control [18], DVD program for strengthening [13], DVD program for walking [22], web-based stretching or toning program [23], and informational handouts about yoga [20]. One of the RCTs compared an in-person yoga intervention with a yoga DVD [19]. All active comparison groups were comparable in dose with the yoga interventions [13,19,22,23]. One of the quasi-experimental studies used health education [16], whereas the other [17] did not describe the comparison group. The methodological quality of the studies ranged from weak (7/12, 58% of studies [14-17,20,21,24]) to moderate (5/12, 42% of studies [13,18,19,22,23]; Table 2).

**Table 2:** General study information, including the first author, type, country, study groups, and results of the quality appraisal assessment.^a^

Author, study type, and country	Study groups	Selection bias	Study design	Confounders	Blinding	Data collection methods	Withdrawals or dropouts	Global rating^b^
Armstrong et al [14], RCT,^c^ United States	Yoga video vs regular activity	Weak	Weak	Weak	Weak	Weak	Weak	Weak
Awdish et al [15], case series, United States	Yoga video; no comparison	Weak	Weak	Weak	Weak	Strong	Weak	Weak
Donesky et al [16], nonrandomized quasi-experimental, United States and United Kingdom	Yoga via videoconferencing vs health education phone call	Weak	Moderate	Weak	Weak	Strong	Moderate	Weak
Gunda et al [17], nonrandomized quasi-experimental, United States	Yoga DVD; control not clearly described	Moderate	Moderate	Strong	Weak	Weak	Moderate	Weak
Huberty et al [18], RCT, United States	Web-based yoga videos vs wait-list control	Moderate	Strong	Strong	Weak	Strong	Strong	Moderate
Huberty et al [23], RCT, United States	Web-based yoga videos (2 doses) vs stretch and tone control	Moderate	Strong	Moderate	Moderate	Strong	Weak	Moderate
Jasti et al [24], single group, India	Tele-yoga module	Moderate	Weak	Weak	Weak	Strong	Weak	Weak
Kyeongra et al [19], RCT, United States	Yoga DVD vs in-person yoga	Strong	Moderate	Strong	Weak	Strong	Moderate	Moderate
Mullur et al [20], RCT, United States	Yoga DVD vs handouts about yoga	Moderate	Weak	Strong	Weak	Moderate	Moderate	Weak
Sakuma et al [21], RCT, Japan	Yoga DVD vs regular activities	Moderate	Strong	Strong	Weak	Strong	Weak	Weak
Schuver et al [22], RCT, United States	Yoga DVD vs DVD on walking	Moderate	Strong	Strong	Moderate	Strong	Moderate	Moderate
Stan et al [13], RCT, United States	Yoga DVD vs DVD on strengthening	Moderate	Strong	Moderate	Weak	Strong	Moderate	Moderate

^a^The quality appraisal assessment was completed using the Quality Assessment Tool for Quantitative Studies with six domains contributing to the score: (1) selection bias, (2) study design, (3) confounders, (4) blinding, (5) data collection methods, and (6) withdrawals and dropouts.

^b^Global ratings were determined as follows: no weak ratings=strong, one weak rating=moderate, and ≥2 weak ratings=weak.

^c^RCT: randomized controlled trial.

### Study Populations

The studies included individuals with physical and mental health conditions, as well as generally healthy adults. For example, 17% (2/12) of studies involved individuals with cancer, such as early-stage breast cancer [13] and myeloproliferative neoplasms (blood cancers) [18]. Approximately 25% (3/12) of studies involved individuals with cardiopulmonary conditions, including pulmonary hypertension [15], neurocardiogenic syncope [17], and a combination of chronic obstructive pulmonary disease (COPD) and heart failure (HF) [16]. Other studies involved sedentary adults who were overweight [19], veterans with diabetes [20], women who had experienced stillbirths [23], and women with depression [22]. Approximately 25% (3/12) of studies involved adults without any specified health conditions, including *adult women* [14], female childcare workers [21], and the general public in India during the COVID-19 pandemic [24]. The mean age of the participants ranged from 21 to 71 years. Across all studies, most participants were female, and 50% (6/12) of studies included only female participants [13-15,21-23]. For more details related to the study populations, refer to Table 3.

**Table 3:** Characteristics of the participants included in the reviewed studies.

Study	Population	Number of Participants				Age (years), mean (SD)				Sex, n (%)					
		Total	Yoga	Control	Total		Yoga	Control	Total			Yoga		Control	
									Female		Male	Female	Male	Female	Male
Armstrong et al [14]	Adult women	30	15	15	55.07 (9.69)		54 (10)	55 (9)	30 (100)		0 (0)	15 (100)	0 (0)	15 (100)	0 (0)
Awdish et al [15]	Women with pulmonary hypertension and additional chronic health conditions	3	3	N/A^a^	48, 32, and 24		48, 32, and 24	N/A	3 (100)		0 (0)	3 (100)	0 (0)	N/A	N/A
Donesky et al [16]	Adults with chronic obstructive pulmonary disease and heart failure	15	7	8	71 (8.5)		73 (14.3)	70.5 (2.7)	10 (66)		5 (33)	4 (57)	3 (43)	6 (75)	2 (25)
Gunda et al [17]	Adults with neurocardiogenic syncope	44	21	23	21 (3)		21 (3)	22 (3)	41 (93)		3 (7)	20 (95)	1 (5)	21 (91)	2 (9)
Huberty et al [18]	Adults with myeloproliferative neoplasm	48	27	21	56.9 (10.3)		58.3 (9.3)	55.0 (11.4)	45 (94)		38 (6)	25 (93)	2 (7)	20 (95)	1 (5)
Huberty et al [23]	Women who have experienced stillbirth	90	30 (YLD^b^) and 30 (YMD^c^)	30	NS^d^		NS	NS	90 (100)		0 (0)	30 (100; YLD) and 30 (100; YMD)	0 (0)	30 (100)	0 (0)
Jasti et al [24]	General adult public	95	95	N/A	40.39 (13.33)		40.39 (13.33)	N/A	69 (73)		26 (27)	69 (73)	26 (27)	N/A	N/A
Kyeongra et al [19]	Sedentary adults who are overweight	14	7	7	58.6 (5.4)		58.7 (4.1)	58.4 (6.8)	12 (86)		2 (14)	6 (86)	1 (14)	6 (86)	1 (14)
Mullur et al [20]	Veterans with CKD^e^ and diabetes	10	5	5	64.4 (NS)		60 (10.34)	68.8 (5.97)	1 (10)		9 (90)	1 (20)	4 (80)	0 (0)	5 (100)
Sakuma et al [21]	Female childcare workers	98	67	31	33.6 (NS)		32.6 (11.5)	35.8 (13.0)	98 (100)		0 (0)	67 (100)	0 (0)	31 (100)	0 (0)
Schuver et al [22]	Women with a history or diagnosis of depression	40	20	20	42.68 (4.95)		45.55 (12.30)	39.8 (11.23)	40 (100)		0 (0)	20 (100)	0 (0)	20 (100)	0 (0)
Stan et al [13]	Women with early-stage breast cancer and cancer-related fatigue	34	18	16	62.1 (8.1)		61.4 (7.0)	63.0 (9.3)	34 (100)		0 (0)	18 (100)	0 (0)	16 (100)	0 (0)

^a^N/A: not applicable.

^b^YLD: yoga low dose (60 min/week).

^c^YMD: yoga moderate dose (150 min/week).

^d^NS: not specified.

^e^CKD: chronic kidney disease.

In addition to extracting information about the enrolled populations, justification of the intervention delivery method based on the enrolled population was extracted when available. This was done to identify whether the study authors chose to implement remote delivery to address population-specific needs. Approximately 50% (6/12) of the studies reported some type of justification for the remote delivery method and related it to population-specific needs. Interestingly, one of these studies was designed during the COVID-19 pandemic and was conducted to provide easily accessible stress management strategies to the general public during social isolation [24]. Approximately 17% (2/12) of the studies [13,16], one for individuals with COPD and HF [16] and the other for women with breast cancer [13], justified their choice of implementing a remote intervention by noting that transportation-related barriers impeded individuals’ abilities to attend in-person interventions. Another study involving veterans with diabetes supported the choice of implementing a remote intervention by noting that physical impairments make it challenging for individuals to access and engage in physical activity [20]. Another study with older adults [14] reported low adherence to in-person exercise interventions in this population. Finally, a study with childcare workers [21] justified remote delivery using a yoga DVD as it was a convenient low-cost option. In contrast, 50% (6/12) of studies [15,17-19,22,23] did not provide any justification for the implementation of remote delivery.

### Yoga Intervention Characteristics

#### Delivery Methods

As per the inclusion criteria, all reviewed studies [13-24] involved remote delivery of yoga. There were no inclusion criteria that stipulated the intervention setting; however, all the included studies stated that the interventions occurred in the participants’ homes. Approximately 83% (10/12) of studies implemented asynchronous delivery through prerecorded yoga videos using DVDs [13,15,17,19-22], a mobile app [15], a website [18,23], and a *video* (type not specified) [14]. Among the studies that delivered yoga via DVDs, one of the studies compared in-person yoga with yoga delivered via DVD [19]. One of the studies implemented synchronous delivery [16] via the videoconferencing platform DocBox (MicroDesign) to provide real-time video interactions between the instructor and the participants. The study team installed the technology in the participants’ homes, and a DocBox technician provided technological support for the entire study duration. One of the studies referred to using a *tele-yoga* module and mentioned *a minimum of supervised* sessions, implying that there were supervised and unsupervised sessions; however, further details of the delivery method were not clearly described [24].

By nature, 83% (10/12) of studies implementing asynchronous delivery were delivered individually [13-15,17-23]. For the study implementing synchronous delivery, the instructor saw the entire group to provide feedback; however, participants only saw the instructor and needed to be unmuted to ask questions [16]. Some studies that implemented asynchronous delivery included interactions with the study team at periodic intervals through phone calls [13,22], in-person visits [14], and introductory classes (one [14,20] or two [19]). Some studies that implemented asynchronous delivery used strategies to optimize safety, including written instructions [13,14,19,20], ability-based movement sequences [15], intensity ramp-ups across videos [18], and preparatory instructions within each video [23]. The study in which the intervention was delivered synchronously included safety measures in which a nurse observed and assessed participants before and after each tele-yoga class [16].

Approximately 50% (6/12) of studies implementing asynchronous delivery used only 1 yoga video or sequence for the study’s duration [13,14,19-22]. In 25% (3/12) of studies, participants used a variety of videos [17,18,23]. One of these studies provided beginner and intermediate ratings to help participants choose videos [18], whereas the other provided 12 videos to be watched in a specific order to optimize safety [17]. In the case series, it was clear that each participant watched different videos; however, it was unclear whether a given participant watched the same yoga video or sequence for the study duration [15]. For the study comparing an in-person yoga intervention with a DVD, it was unclear whether the in-person sessions differed from week to week and whether the in-person and DVD groups performed the same sessions [19].

#### Yoga Intervention Components and Other Details

Approximately 50% (6/12) of studies did not specify the style of yoga used [13,14,17,20,21,24], whereas 50% (6/12) used specific styles, including gentle *Hatha* yoga [22,23], *Vinyasa* yoga [19], *Iyengar* yoga [16], a combination of *Hatha* yoga and *Iyengar* [15], and a combination of *Hatha* yoga and *Vinyasa* [18]. Approximately 50% (6/12) of studies [15,16,20,22-24] indicated that the yoga interventions were designed or adapted for their specific populations. One of these studies also provided additional preselected videos available on the web for participants to choose from after completing the videos designed for the intervention [23]. Approximately 17% (2/12) of studies [13,19] indicated that the yoga videos were selected with the population in mind. Approximately 17% (2/12) of studies [14,17] did not mention whether the programs were chosen for their populations. One of the studies used a yoga program that had previously shown benefits in people with low back pain; however, further rationale for its selection was not provided [21]. One of the studies selected specific yoga videos posted on the web but also created 6 videos specifically for the study [23].

Approximately 42% (5/12) of studies [14,17,20-22] did not specify the credentials of yoga instructors. One of the studies [13] specified that a certified yoga therapist provided the intervention, whereas another [16] specified that a certified yoga instructor and physical therapy assistant provided the intervention. The remaining studies specified that certified yoga instructors (500-hour certifications [18] or >200-hour certifications [19,23]) provided the interventions.

Approximately 33% (4/12) of studies specified using breathing exercises and physical postures [19-21,24]. One of the studies specified using postures and meditation [23]. Approximately 58% (7/12) of studies mentioned using breathing exercises, postures, and meditation and relaxation [13-16,18,22]. Of these 7 studies, 4 (57%) used meditation [15,17,18,22], 2 (29%) used relaxation [13,14], and 1 (14%) used both relaxation and meditation [16].

The yoga interventions ranged in duration from 2 to 12 weeks, with individual session lengths ranging from 10 to 90 minutes and frequency ranging from once a week to daily practice. Interestingly, one of the studies included 2 different prescribed yoga doses (low, 60 min/week, and moderate, 150 min/week) [23].

Most studies considered remotely delivered interventions as home practice and, therefore, did not assess or account for additional home practice. However, they accounted for all the practices at home when reporting adherence (if reported). The study comparing in-person yoga with a yoga DVD specified instructions for the desired frequency of additional home practice [19]. See Table 4 for a summary of the yoga intervention characteristics.

**Table 4:** Information about intervention characteristics.^a^

Study	Asynchronous vs synchronous	Technology	Group vs individual	Yoga style	Yoga limbs	Duration (minutes per session)	Sessions per week	Number of weeks
Armstrong et al [14]	Asynchronous	Video (type unspecified)	Individual	NS^b^	Breathing, postures, and relaxation	30	4	10
Awdish et al [15]	Asynchronous	DVD and mobile app	Individual	Hatha and Iyengar	Breathing, postures, and meditation	NS	3 to 6	8
Donesky et al [16]	Synchronous	Videoconference	Group	Iyengar	Breathing, postures, meditation, and relaxation	60	2	8
Gunda et al [17]	Asynchronous	DVD	Individual	NS	Breathing, postures, and relaxation	60	3	12
Huberty et al [18]	Asynchronous	Web-based videos	Individual	Hatha and Vinyasa	Breathing, postures, and meditation	Requested 60 min/week	Requested 60 min/week	12
Huberty et al [23]	Asynchronous	Web-based videos	Individual	Gentle Hatha	Postures and meditation	60 min week (LD^c^); 150 min/week (HD^d^)	60 min/week (LD); 150 min/week (HD)	12
Jasti et al [24]	Asynchronous and synchronous	Unspecified tele-yoga	NS	NS	Breathing and postures	40	≥1	4
Kyeongra et al [19]	Asynchronous	DVD	Individual	Vinyasa	Breathing and postures	Required one 90-minute session, encouraged 2 more for “home practice”	Required one 90-minute session, encouraged 2 more for “home practice”	8
Mullur et al [20]	Asynchronous	DVD	Individual	NS	Breathing and postures	10	As often as possible	12
Sakuma et al [21]	Asynchronous	DVD	Individual	NS	Breathing and postures	7.5	Daily	2
Schuver et al [22]	Asynchronous	DVD	Individual	Gentle Hatha	Breathing, postures, and meditation	60 to 75	2	12
Stan et al [13]	Asynchronous	DVD	Individual	NS	Breathing and postures	90	3 to 5	12

^a^Information about the intervention characteristics such as the delivery method, including whether the intervention was delivered synchronously or asynchronously; the type of technology used; whether the intervention was delivered to a group or individual; and yoga intervention components, including the yoga style, yoga limbs, and intervention dose (frequency and duration).

^b^NS: not specified.

^c^LD: low dose.

^d^HD: high dose.

### Intervention Feasibility and Safety

#### Overview

The extracted data that were related to feasibility included information about adherence and occurrence or absence of technological challenges. The components of adherence were subcategorized into intervention adherence (ie, whether participants completed the intended yoga intervention dose) and study adherence (ie, whether participants completed the study overall). The following sections present the results related to adherence. We reported the results as specified in each study and used the term *compliance* instead of adherence for one of the studies that reported its results using that term.

#### Intervention Adherence

Adherence to the intervention was assessed or reported differently across studies. Approximately 33% (4/12) of studies did not report on intervention adherence [14,15,17,20]. Approximately 25% (3/12) of studies [13,21,23] defined benchmarks or categories such as *compliance* or *good adherence* using specific thresholds. Approximately 42% (5/12) of studies reported intervention adherence through mean yoga practice using self-reported logs [18,19,22,23] or class attendance (synchronous [16] and in-person [19] interventions). In addition to self-reporting, 17% (2/12) of studies using web-based videos also used web analytics programs to monitor the time spent viewing these videos [18,23].

One of the studies set a benchmark for adherence (completion of 90% of the prescribed yoga dose in 9 out of 12 weeks). In this study, there was a low-dose group, in which 44% (8/18) of the participants met the benchmark, and a moderate-dose group, in which 6% (1/16) met the benchmark [23]. The study using the term *compliance* defined it as exercising >3 times per week for ≥7 weeks [13]. The authors showed that 39% (7/18) of the yoga group were compliant with the intervention as compared with 44% (7/16) in the strengthening group [13]. Finally, another study defined good adherence as those who practiced ≥6 times over 2 weeks and defined poor adherence as those who practiced 1 to 5 times over 2 weeks [21]. This study showed that 55% (37/67) of the yoga group had good adherence, 16% (11/67) had poor adherence, whereas 10% (7/67) did not report their adherence [21].

The mean yoga practice was also reported in several studies to indicate yoga intervention adherence. In the study comparing a yoga DVD with a walking DVD, a mean practice of 119.75 (SD 58.95) minutes for the yoga group was reported and a mean practice of 78.25 (SD 52.50) was reported for the walking group [22]. The target intervention dose for both groups was 120 minutes [22]. The study comparing in-person yoga with yoga delivered via a DVD reported that the in-person group practiced yoga for 75 minutes per week compared with 53.4 minutes per week in the DVD group [19]. The targeted dose was administered for 90 minutes per week. One of the studies using web-based videos showed mean yoga participation of 40.8 minutes per week by use of a software that counted how long they viewed web-based videos [18]. However, the self-reported mean practice of the same participants was 56 min/week. However, based on the self-reported practice measure, only 15% (4/27) of participants completed the required intervention dose of ≥60 minutes per week [18]. The other study using web-based videos showed, via self-report, that the low-dose group achieved a mean weekly yoga practice of 73% (44/60 minutes) of the target dose, and the moderate-dose group achieved a mean weekly yoga practice of 49% (77/150 minutes) of the prescribed dose [23]. However, web analytics revealed that these numbers overreported yoga practice [23]. Another study reported a mean yoga practice of 11.48 (SD 7.55) sessions but did not indicate a target dose [24]. Finally, the synchronous yoga study showed a mean attendance of 90% (14.5/16 required yoga classes) [16].

#### Study Adherence

In addition to intervention adherence, study adherence was reported as the number of individuals who completed the study compared with those who were enrolled. Approximately 33% (4/12) of studies did not report whether any individuals dropped out or whether all those enrolled completed the study [14,15,17,20]. In the study comparing yoga DVDs with a control group, 66% (44/67) of participants in the yoga group and 77% (24/31) of participants in the control group completed the study [21]. In the study comparing a yoga DVD with a strengthening DVD, 78% (14/18) of participants in the yoga group and 56% (9/16) of participants in the strengthening group completed the study [13]. The study comparing a yoga DVD with a walking program DVD reported that 90% (18/20) of participants in the yoga group and 80% (16/20) of participants in the walking group completed the study [22]. The study comparing yoga delivered via videoconferencing with an educational phone call control reported that 86% (6/7) of participants in the yoga group and 75% (6/8) of participants in the control group completed the study [16]. The study comparing in-person yoga with DVD yoga showed that 86% (6/7) of participants in the in-person group and 57% (4/7) of participants in the DVD group completed the study [19]. The study comparing web-based yoga videos with a wait-list control group reported that 79% (27/34) of participants in the yoga group and 75% (21/28) of participants in the control group completed the study [18]. The study comparing web-based yoga videos with a stretch and tone program reported that 57% (34/60) of participants in the yoga group and 47% (14/30) of participants in the control group completed the study [23]. Finally, the single-group study reported that 57% (54/95) of participants completed the study [24].

#### Technical Challenges and Satisfaction

The study implementing synchronous yoga reported technological challenges in 77% (24/31) of the yoga sessions and a mean enjoyment of 8.3 (SD 2.7) on a 10-point scale [16]. One of the studies using web-based videos reported that participants noted some technological challenges, attributing most to slow internet connections [23]. This study also noted high participant satisfaction in the web-based yoga video group and the web-based tone and stretch group [23]. The single-group study involving an unspecified tele-yoga program reported that 92.6% of the participants reported the intervention to be feasible (and safe) [24]. Finally, one of the studies assessed program satisfaction and found that the in-person group showed significantly greater satisfaction with the instruction method than the DVD group [19].

#### Adverse Events and Safety

Approximately 33% (4/12) of studies did not specify the occurrence or absence of adverse events [14,17,19,22]. Approximately 42% (5/12) of studies reported no adverse events [15,16,18,20,21]. One of the studies reported that 92.6% of the participants reported the intervention to be safe and feasible but did not provide further details [24]. One of the studies comparing a yoga DVD program with a strengthening DVD program for women with breast cancer reported 4 mild adverse events in the strengthening group and 9 mild adverse events in the yoga group [22]. However, the authors concluded that these mild adverse events could be attributed to recent reconstructive surgeries or medication side effects related to the participants’ cancer treatment and management [22].

### Preliminary Efficacy

#### Patient-Reported Outcome Measures

Patient-reported outcomes included various measures assessing anxiety, depression, sleep, fatigue, quality of life, general health, syncope functional status, multifactorial myeloproliferative neoplasm symptoms, sexual function, and pain. One of the studies did not analyze whether there were statistically significant differences between pre- and postmeasurements, despite having collected the data [18]. The single-group study showed significant improvements in perceived stress and yoga performance [24]. The study comparing a yoga DVD with a walking program DVD for women with depression showed within-group improvements but did not show between-group differences [22]. However, when controlling for baseline levels of rumination, the study showed significantly lower rumination in the yoga group [22]. The study comparing the use of a yoga DVD with a strengthening DVD showed significant within-group improvements in fatigue but did not show significant between-group differences [13]. The study that included childcare workers showed statistically significant improvements in low back pain, upper arm or neck pain, and menstrual pain in individuals who demonstrated good yoga intervention adherence [21]. The study comparing a web-based stretch or tone program with a web-based yoga program for women who had experienced stillbirth noted significant improvements in depression, perinatal grief, self-compassion, and self-rated health, favoring the yoga group [23]. Finally, the study that included individuals with neurocardiogenic syncope showed a statistically significant improvement on the Syncope Functional Status questionnaire following the yoga intervention [17].

#### Physical Performance and Function Outcome Measures

Physical performance and functional outcome measures comprised flexibility, strength, the 6-minute walk test, and balance. One of the studies assessing the effect of yoga on flexibility in older women showed statistically significant improvements in the sit and reach test in the yoga group, along with improvements in trunk extension, shoulder flexion, and left and right ankle flexibility [14]. In contrast, another study investigating the impact of a yoga DVD on childcare workers did not show any improvements in flexibility [21]. However, they did not specify the measure of flexibility used [21]. The studies assessing upper and lower extremity strength [16] and assessing grip strength [21] did not show any improvements. Approximately 17% (2/12) of studies [15,16] found no improvements in the 6-minute walk test; however, one of these studies [16] showed statistically significant improvements in shortness of breath and distress related to dyspnea in the yoga group following the 6-minute walk test. The study assessing balance used the functional reach test and did not report any improvements [21].

#### Physiological Outcome Measures

Physiological measures that showed significant improvements in at least one study included heart rate, blood pressure, oxygen saturation, presyncope and syncope events, and specific blood tests. One of the studies on veterans with diabetes showed statistically significant improvements in heart rate, diastolic blood pressure, and capillary blood glucose [20], whereas another study on individuals with neurocardiogenic syncope did not show any significant improvement in heart rate or blood pressure [17]. The study on individuals with neurocardiogenic syncope also showed statistically significant improvements in the number of presyncope and syncope events in participants who completed the yoga program [17]. See Table 5 for a summary of the results of each study for the patient-reported outcome measures, physical performance and performance outcome measures, and physiological measures.

**Table 5:** Outcome measures assessed in each study.

Study and type	Comparison	Outcomes
Armstrong et al [14], RCT^a^	Yoga video vs regular activity	Sit and reach test^b^ Trunk extension^b^ Trunk flexion Shoulder extension Shoulder flexion^b^ Left ankle flexibility^b^ Right ankle flexibility^b^
Awdish et al [15], case series	Yoga video; no comparison	Subjective changes via journaling^c^ Health Promoting Lifestyle Profile 2^c^ 6-minute walk test^c^ Oxygen saturation^c^
Donesky et al [16], quasi-experimental nonrandomized study	Yoga via videoconferencing vs health education phone call	Safety: see the Adverse Events and Safety section Acceptability: see the Intervention Adherence section Technical issues: see the Patient-Reported Outcome Measures section Upper and lower body muscle strength 6-minute walk test Symptoms following the 6-minute walk test^b^ Quality of life: St George’s Respiratory Questionnaire and Kansas City Cardiomyopathy Questionnaire^d^ Depression Personal Health Questionnaire-8 Overall dyspnea: Dyspnea-12 questionnaire General Sleep Disturbance Scale
Gunda et al [17], quasi-experimental nonrandomized pilot study	Yoga DVD; control not clearly described	Log of the number of presyncope and syncope events: in the intervention group, for those who finished the yoga regimen, there was a statistically significant improvement in the number of episodes of syncope and presyncope SFSQ^e^: statistically significant decrease in the mean SFSQ score from the control phase to completion of the intervention phase Head-up tilt table: resting heart rate Blood pressure
Huberty et al [18], pilot RCT	Web-based yoga videos vs wait-list control	Yoga participation: see the Intervention Adherence section Adverse events: see the Adverse Events and Safety section Blood draw feasibility and practicality Inflammatory biomarkers^c^ Fatigue: single item from the multifactor MPN-SAF^c,f^ Multifactor MPN-SAF: total symptom score^c^ Quality of life: single item from the NIH^g^ PROMIS^h^ Global Health measure^c^ Sleep disturbance: Sleep Disturbance Scale Short Form 8a^c^ Pain intensity: Pain Intensity Short Form 3a^c^ Anxiety distress: Emotional Anxiety Short Form 8a^c^ Depression emotional Distress: Depression Short Form 8a^c^ Mental health: PROMIS^c^ Sexual function: 8-item for men; 10-item for women^c^ Physical health: PROMIS^c^
Huberty et al [23], RCT	Web-based yoga videos, including 2 different doses vs stretch and tone control	Adherence: see the Intervention Adherence section Acceptability: all groups achieved satisfaction benchmarks Adverse events: see the Adverse Events and Safety section Demand: no group met the demand benchmark Impact of Event Scale State-trait Anxiety Inventory Patient Health Questionnaire-9^i^ Perinatal Grief Scale^i^ Self-Compassion Scale^i^ Self-rated health (Short Form-12)^i^: a significant decrease in low-dose and control groups Emotion Regulation Questionnaire Mindful Attention Awareness Scale Pittsburg Sleep Quality Index
Jasti et al [24], single-group open-label trial	Tele-yoga module	Adherence: see the Intervention Adherence section Difficulty rating: mean score indicated that the yoga module was easy to practice Feasibility: 92.6% of participants found the yoga to be safe and feasible Yoga Performance Assessment scale^d^ Perceived Stress Scale^d^
Kyeongra et al [19], pilot RCT	Yoga DVD vs in-person yoga	Adherence: see the Intervention Adherence section Modifiable Activity Questionnaire Program satisfaction: see the Patient-Reported Outcome Measures section
Mullur et al [20], pilot RCT	Yoga DVD vs handout about yoga	Capillary blood glucose^b^ Heart rate^b^ Diastolic blood pressure^b^ Hemoglobin A1c Systolic blood pressure Weight BMI
Sakuma et al [21], RCT	Yoga DVD vs regular activities	Measure of body pain according to the Visual Analog Scale (range 0-100)^j^ Japanese version of the General Health Questionnaire Body weight BMI Flexibility (measure not described reported in comparator) Grip strength Functional reach test
Schuver et al [22], pilot RCT	Yoga DVD vs walking DVD	Beck Depression Inventory^d^ Ruminative Responses Scale^k^
Stan et al [13], pilot RCT	DVD vs strengthening DVD	Feasibility: see the Intervention Adherence section Safety: see the Adverse Events and Safety section Fatigue: Multidimensional Fatigue Symptom Intervention Short Form^d^ Quality of life: Functional Assessment of Cancer Therapies–Breast^d^

^a^RCT: randomized controlled trial.

^b^Statistically significant between-group difference favoring the remote-delivered yoga group.

^c^Only effect sizes were calculated.

^d^Statistically significant within-group differences.

^e^SFSQ: Syncope Functional Status Questionnaire.

^f^MPN-SAF: Myeloproliferative Neoplasm Symptom Assessment Form.

^g^NIH: National Institutes of Health.

^h^PROMIS: Patient-Reported Outcomes Measurement Information System.

^i^Statistically significant improvements in the yoga group compared with the control group. Yoga comprised 2 different yoga intervention doses. For further details, refer to the study by Huberty et al [23].

^j^Statistically significant improvement for individuals who demonstrated good adherence to the yoga group (≥6 times per 2 weeks) for low back pain, upper arm or neck pain, and menstrual pain.

^k^Statistically significant between-group difference when controlling for baseline levels.

## Discussion

### Principal Findings and Comparison With Prior Work

The purpose of this scoping review was to examine the existing literature regarding the practice of yoga through remote delivery methods and identify current gaps related to (1) the populations studied, (2) the intervention characteristics (delivery methods and intervention components implemented), (3) the safety and feasibility of the interventions, and (4) the preliminary efficacy of the interventions. In summary, the studied populations included adults across their life spans, including individuals with physical and mental health conditions and some generally healthy adults. The review showed that, to date, most studies implementing remotely delivered yoga have implemented asynchronous delivery. In addition, the delivered interventions were primarily *Hatha yoga* interventions, including postures, breathing exercises, and meditation and relaxation exercises. The interventions were shown to be generally safe and feasible, with some feasibility challenges present in the study that implemented synchronous delivery. The heterogeneity of the included studies did not allow for an adequate evaluation of the preliminary efficacy of remotely delivered yoga interventions.

This scoping review showed that remotely delivered yoga has been successfully implemented in a heterogeneous sample of populations with and without chronic conditions. This is similar to the body of literature investigating the impact of in-person yoga [6]. In this review, studies included older adults; childcare workers; and individuals with chronic conditions such as COPD, HF, cancer, depression, diabetes, pulmonary hypertension, and cardiogenic syncope. Notably, most of these populations have also been implicated in in-person yoga studies. For instance, individuals with COPD [25] and HF [26], individuals with cancer [27,28], women with depression [29], older adults [30], adults with diabetes [31], and adults with cardiovascular conditions [32] have all been included in previous in-person yoga studies. Interestingly, no two studies reviewed here were conducted on the same population. Replication of studies for similar populations is seen in the in-person yoga literature [6] but is missing from the body of literature reviewed in this study. Therefore, replication using larger samples is needed. In addition, despite the variety of populations enrolled in the included studies, some populations that were commonly enrolled in in-person yoga studies were not included in the reviewed studies. For example, none of the included studies involved populations of individuals with neurological conditions or balance impairments. This differs from the body of literature investigating in-person yoga, which shows a large body of studies investigating yoga in these individuals [33,34].

It is unclear why some populations were enrolled in studies investigating remotely delivered yoga, whereas others were not. In an attempt to gain a better understanding of this, information related to the authors’ motivation to implement remote delivery was extracted. However, as reported in the *Results* section, half of the studies (6/12, 50%) [15,17-19,22,23] did not provide any reason for choosing the remote delivery method. Previous guidelines for the development of yoga interventions have highlighted that intervention delivery must be considered in intervention design and are discussed in the second domain of *dose and delivery of yoga* [35]. There are multiple reasons why remote delivery may be beneficial, some of which have been mentioned in the reviewed studies. For example, previous studies have shown that multiple factors can impede engagement in exercise and physical activity, including transportation barriers, lack of time, decreased motivation, feelings of intimidation because of physical or environmental barriers, costliness, stigma, and a general lack of resources [36-38]. These barriers may also impede access to in-person yoga, and as such, a better understanding of facilitators and barriers associated with in-person and remote delivery methods is needed. In fact, previous studies support the notion that interventions delivered through technology show higher adherence in older adults [39].

When examining delivery method characteristics, most studies (10/12, 83%) implemented asynchronous delivery. This is similar to the results of another scoping review that investigated web-based mindfulness interventions for people with physical health conditions, which showed that 69% (11/16 studies) implemented asynchronous delivery [40]. Furthermore, another systematic review investigating remotely delivered therapy for mental health showed that 73% (8/11 studies) implemented asynchronous delivery [41]. This may be because asynchronous delivery requires fewer resources; however, this has not been formally established. Specifically, for the purpose of this review, no studies to date have compared synchronous and asynchronous remote delivery methods in yoga interventions. A total of 2 previous reviews also showed that there were no studies comparing synchronous and asynchronous remote delivery methods for their respective interventions: web-based mindfulness [40] and remotely delivered therapy [41]. One of the studies comparing synchronous and asynchronous delivery of tele-exercise for individuals with spinal cord injury showed significantly higher adherence and average weekly training load for the synchronous training group compared with the asynchronous group, suggesting that synchronous training may offer added benefits [42]. Thus, a current gap in the literature relates to the investigation of synchronous remote delivery of yoga, despite promising outcomes for other interventions [40-42].

Although more studies included in our review implemented asynchronous delivery than synchronous delivery, the one study investigating synchronous delivery reported high adherence and enjoyment [16]. However, it also showed some feasibility challenges. It should be acknowledged that we cannot arrive at conclusions about the feasibility of all synchronously delivered, remotely delivered yoga interventions as there was only one study included in this review that implemented synchronous delivery. In addition, the other scoping reviews that investigated remotely delivered interventions did not speak to the feasibility of synchronously delivered interventions [40,41]. A previously published study indicated that certain videoconferencing platforms and practices may facilitate web-based engagement in remote research [43]. Thus, synchronous yoga interventions using widely available and commonly used videoconferencing platforms should be implemented and investigated to determine the approaches that limit technological challenges.

When examining the other intervention characteristics, such as the implemented style and limbs of yoga, our findings are similar to those found in the in-person yoga literature [44]. Although some of the studies reviewed here did not report the yoga style or limbs used, others reported a style of yoga that fell under the umbrella of *Hatha yoga* (the physical practice of yoga) without further specification. Limited available guidance on the optimal style may be is the reason behind a lack of specific reporting surrounding yoga style in general. In fact, a previously published systematic review [45] indicated that RCTs using different yoga styles did not differ significantly in their odds of achieving their desired outcomes. This demonstrated that there may not be an optimal style of yoga, and other factors such as preference and availability can help determine style [45]. However, future work should strive to report the style and limbs of yoga being implemented in interventions to help identify whether there are population-specific intervention characteristics that can optimize outcomes. For instance, a specific yoga style may be more beneficial for a given population. For example, a study comparing meditative yoga to power yoga for stress reduction in physically active, yoga-naive women showed significant improvements in salivary cortisol and state anxiety following a single meditative yoga session compared with power yoga [46], demonstrating that certain types of yoga may be more beneficial in specific situations. In addition, one yoga style may be delivered remotely more easily than another. For example, it may be more challenging for individuals with chronic health conditions and limited yoga experience to engage in a *Vinyasa* flow intervention involving rapid transitions from posture to posture while trying to follow a prerecorded video. However, this has yet to be investigated and could not be examined in this review or previously published reviews [6,45]. Thus, future studies should investigate the interactions among yoga style, study population, and other intervention characteristics, especially delivery method.

### Limitations

This scoping review had some limitations. First, only studies with pre- and posttesting were included. This was stipulated in the eligibility criteria and was intended to facilitate the exploration of preliminary efficacy. However, this may have resulted in the exclusion of studies, such as feasibility studies or qualitative analyses, which could have provided additional insight into the current state of the literature. Specifically, we are aware of 4 studies [47-50] that were excluded because of a lack of explicit pre- and posttesting. Second, the heterogeneity of the included studies and assessed outcomes prevented the identification of conclusive preliminary efficacy findings. Third, the searches were completed in April 2021. With the continuation of the COVID-19 pandemic, publications involving telerehabilitation in general [51-53] and remotely delivered yoga interventions, in particular, may increase over the next few years. This could possibly require an update to this review. Regardless of these limitations, this scoping review identifies key gaps in the related literature and provides a strong foundation to optimize future research.

### Strengths

This scoping review had several strengths. A broad range of databases was searched, which allowed a comprehensive search. This review provides a robust quality assessment of the included studies, provides a realistic picture of the literature and facilitates the interpretation of the findings. In addition, this review covers a broad range of content areas, including (1) the populations studied, (2) the intervention characteristics (delivery methods and intervention components implemented), (3) the safety and feasibility of the interventions, and (4) the preliminary efficacy of the interventions. This broad range of content allows readers to obtain a full picture of the state of the related literature and understand the current gaps. This is intended to help provide a path forward to optimize future research.

### Future Directions

Multiple steps can be taken to address the gaps identified in this review and optimize future research. Future studies involving larger sample sizes should assess populations similar to those enrolled in the reviewed studies to determine whether the results can be replicated. In addition, populations not examined in the included studies, such as those with neurological conditions or other populations that have been shown to benefit from in-person yoga, should be enrolled in future studies that implement remotely delivered yoga. Future studies should justify the choice of delivery methods and relate this justification to population-specific needs. Moreover, future studies should consider and investigate the interactions among delivery methods, yoga intervention components, and other study characteristics. They should explore the implementation of synchronous delivery and compare different delivery methods. Specifically, synchronous yoga interventions using widely available and commonly used videoconferencing platforms should be investigated to determine whether this approach limits technological challenges and facilitates feasibility. Finally, future studies should report information regarding adverse events, adherence, and other safety and feasibility measures to provide robust information regarding the implementation of these interventions.

### Conclusions

This review synthesized the literature regarding the remote delivery of yoga and provided information about gaps in the literature related to study populations, intervention characteristics, intervention safety and feasibility, and intervention efficacy. Overall, this review revealed a broad gap in the literature, showing that little attention has been paid to yoga intervention delivery methods. Future studies and yoga intervention development guidelines should further consider the delivery methods when developing interventions. For instance, population-specific needs and barriers should be accounted for when determining delivery methods. In addition, more studies implementing synchronous delivery methods and studies comparing delivery methods should be conducted, and robust reporting of intervention characteristics is required.

## Appendix

Multimedia Appendix 1 The search strategy used for this scoping review, including the search terms used for each database, along with notes detailing the search methods.

## Abbreviations

COPD: chronic obstructive pulmonary disease
HF: heart failure
PRISMA: Preferred Reporting Items for Systematic Reviews and Meta-Analyses
PRISMA-ScR: Preferred Reporting Items for Systematic Reviews and Meta-Analyses extension for Scoping Reviews
RCT: randomized controlled trial
