# Supplementary material for: Remote Delivery of Yoga Interventions Through Technology: Scoping Review
Source: J Med Internet Res. 2022 Jun 6;24(6):e29092. doi: 10.2196/29092 (PMC9210204; doi:10.2196/29092)
Supplement: Multimedia Appendix 1 [file jmir_v24i6e29092_app1.pdf]

Table S1. Search Strategy

Database	Search Terms
CINAHL	(yoga OR Iyengar OR ashtanga OR hatha OR asana) AND (telerehabilitation OR tele-rehabilitation OR telemedicine OR videoconferencing OR video OR telenursing OR DVD OR remote delivery OR eHealth OR video games OR virtual reality)<br>NOTES: *Used one line for all yoga terms and one line for all telerehabilitation terms joined with "AND," no fields, filters, or limits selected*
Cochrane	(yoga OR Iyengar OR ashtanga OR hatha OR asana) AND (telerehabilitation OR tele-rehabilitation OR telemedicine OR videoconferencing OR video OR telenursing OR DVD OR remote delivery OR eHealth OR video games OR virtual reality)<br>NOTES: *Used All Text field, no limits, or filters*
IEEE Xplore	(yoga OR Iyengar OR ashtanga OR hatha OR asana) AND (telerehabilitation OR tele-rehabilitation OR telemedicine OR videoconferencing OR video OR telenursing OR DVD OR remote delivery OR eHealth OR video game OR virtual reality)<br>NOTES: *Used main search bar with the field All, no limits or filters*
PEDro	Yoga, video<br>Tele*, yoga<br>Yoga, online<br>Yoga, virtual reality<br>Yoga, DVD<br>NOTES: *Used Simple Search with each combination noted above (PEDro does not use Boolean operators), no limits or filters*
PsycInfo	(yoga OR Iyengar OR ashtanga OR hatha OR asana) AND (telerehabilitation OR tele-rehabilitation OR telemedicine OR videoconferencing OR video OR telenursing OR DVD OR remote delivery OR eHealth OR video game OR virtual reality))<br>NOTES: *Used field for Keyword no limits or filters selected*
PubMed	("Yoga"[Mesh] OR yoga OR Iyengar OR ashtanga OR hatha OR asana) AND (telerehabilitation"[Mesh] OR "Telemedicine"[Mesh] OR "Videoconferencing"[Mesh] OR "Video Recording"[Mesh] OR "Telenursing"[Mesh] OR "Video Games"[Mesh] OR "Virtual Reality"[Mesh] OR tele-rehabilitation OR telemedicine OR videoconferencing OR video OR telenursing OR DVD OR remote delivery OR eHealth OR video games OR virtual reality)<br>NOTES: *Used main search bar, no limits, filters, or fields selected*
Scopus	(yoga OR Iyengar OR ashtanga OR hatha OR asana) –all fields AND (telerehabilitation OR tele-rehabilitation OR telemedicine OR videoconferencing OR video OR telenursing OR dvd OR remote AND delivery OR eHealth OR video game OR virtual reality)—all fields<br>NOTES: *Used Advanced Search and one line for all yoga terms and one line for all telerehabilitation terms joined with "AND", no limits or filters*
Web of Science (topic)	(yoga OR Iyengar OR ashtanga OR hatha OR asana) AND (telerehabilitation OR tele-rehabilitation OR telemedicine OR videoconferencing OR video OR telenursing OR DVD OR remote delivery OR eHealth OR video games OR virtual reality)<br>NOTES: *Used All field, no limits, filters*
